# Kin discrimination via odour in the cooperatively breeding banded mongoose

**DOI:** 10.1098/rsos.171798

**Published:** 2018-03-28

**Authors:** J. Mitchell, S. Kyabulima, R. Businge, M. A. Cant, H. J. Nichols

**Affiliations:** 1 School of Natural Sciences and Psychology, Liverpool John Moores University, Liverpool, UK; 2 Banded Mongoose Research Project, Mweya, Queen Elizabeth National Park, Uganda; 3 Centre for Ecology and Conservation, University of Exeter, Penryn Campus, Cornwall, UK

**Keywords:** inbreeding avoidance, scent communication, kin recognition, cooperative breeder, relatedness

## Abstract

Kin discrimination is often beneficial for group-living animals as it aids in inbreeding avoidance and providing nepotistic help. In mammals, the use of olfactory cues in kin discrimination is widespread and may occur through learning the scents of individuals that are likely to be relatives, or by assessing genetic relatedness directly through assessing odour similarity (phenotype matching). We use scent presentations to investigate these possibilities in a wild population of the banded mongoose *Mungos mungo*, a cooperative breeder in which inbreeding risk is high and females breed communally, disrupting behavioural cues to kinship. We find that adults show heightened behavioural responses to unfamiliar (extra-group) scents than to familiar (within-group) scents. Interestingly, we found that responses to familiar odours, but not unfamiliar odours, varied with relatedness. This suggests that banded mongooses are either able to use an effective behavioural rule to identify likely relatives from within their group, or that phenotype matching is used in the context of within-group kin recognition but not extra-group kin recognition. In other cooperative breeders, familiarity is used within the group and phenotype matching may be used to identify unfamiliar kin. However, for the banded mongoose this pattern may be reversed, most likely due to their unusual breeding system which disrupts within-group behavioural cues to kinship.

## 1. Introduction

Breeding between close relatives is well known to entail a fitness cost, known as inbreeding depression, which can manifest in forms such as reduced survival, growth and reproductive success of inbred individuals [1]. It is therefore not surprising that inbreeding avoidance is commonplace in the animal kingdom [2]. One of the most frequent forms of inbreeding avoidance involves either one or both sexes dispersing away from the family group at sexual maturity [3–5]. However, among cooperative breeders, dispersal is usually delayed until after sexual maturity as offspring remain with their parents and help to rear subsequent broods or litters [6,7]. Social groups therefore often consist of close relatives with the potential to inbreed [8]. Thus, such species must use alternative mechanisms of inbreeding avoidance that involve discriminating against kin as mates [9,10].

There are two main mechanisms that group-living animals might use to discriminate levels of relatedness within and between groups. First, as the degree of association between individuals usually varies with kinship, it is often possible for individuals to assess the likely level of relatedness between themselves and others through associative learning of social familiarity [11]. Assessing potential levels of kinship based on familiarity (usually learned at the juvenile stage) appears to be both widespread and effective among cooperative breeders [12,13]. For example, in Seychelles warblers *Acrocephalus sechellensis* and western bluebirds *Sialia mexicana*, cross-fostering experiments have shown that young learn the calls of individuals that tended them in the nest and treat them as relatives, regardless of actual levels of relatedness [14,15].

As an animal may be familiar with individuals that vary in kinship levels, social familiarity is usually combined with simple rules to gauge likely levels of relatedness. For example, although an individual will be familiar with all members of its group, it may only be willing to mate with individuals that immigrated into the group after it was born and hence are unlikely to be relatives [16,17]. Similarly, dominant males may avoid inbreeding with their daughters by employing the rule ‘avoid breeding with females born into the group during dominance tenure’ [18], or ‘avoid breeding with the daughters of previous mates’ [19]. Similar rules may exist that do not necessarily require differences in familiarity with the individuals concerned, for example, individuals may avoid mating with others of age groups likely to contain close relatives [19] or in locations that may contain relatives [20–22].

The second mechanism by which animals may avoid inbreeding involves using direct cues to genetic relatedness, for example through phenotype matching [23,24]. Here, individuals discriminate between different degrees of relatives based on odour similarity to themselves. Such discrimination is likely to be facilitated through prenatal exposure to odorants from an individual's own (and maternal/sibling) metabolites that influence the development of olfactory sensory neurones (OSN) [25]. Individuals should therefore develop enhanced detection and discrimination of odours associated with relatives. Evidence that relatedness is detected through phenotype matching has rarely been found in cooperative breeders, perhaps as close relatives can usually be determined through familiarity [10]. However, in cooperatively breeding meerkats, scent-presentation experiments demonstrated that dominant females are able to discriminate between the anal gland odours of males of high and low relatedness, even when they are unfamiliar with the males [26]. Similarly, in cooperatively breeding cichlids, individuals choose to associate with others based on chemical cues to relatedness [27]. Similar patterns have also been shown in species that rear broods of mixed parentage where relatedness may be difficult to detect based on familiarity, such as the bluegill sunfish *Lepomis macrochirus* [28] and the house mouse *Mus musculus domesticus* [29].

The banded mongoose *Mungos mungo* provides an excellent opportunity to investigate the use of scent cues in identifying kin. This African mammal lives in mixed sex groups (mean group size = 29 individuals), that contain a ‘core’ of 1–5 dominant breeders of each sex that breed up to four times per year, alongside younger subordinates that breed occasionally [30]. Breeding is highly synchronized within but not between groups, with all female group-members coming into oestrus within a week of each other and giving birth together in tight synchrony, on the same night in over 60% of cases [31]. The litters of individual females are combined into a large communal litter immediately after birth. Previous research suggests that tight birth synchrony disrupts cues to the pups' parentage and hence reduces infanticide by dominant females, who risk killing their own pups. When births occur asynchronously (or when dominant females are experimentally treated with contraceptives), dominant females are able to identify the pups of other females and levels of infanticide increase substantially [31,32]. Litters are raised communally by the group with both breeders and non-breeders contributing to care [30] and helpers and pups do not assort by relatedness, but instead helping is biased toward same-sex offspring [33].

In the banded mongoose, kin discrimination does not appear to occur at the pup stage (although this remains to be explicitly tested) yet there is evidence that kin discrimination occurs among adults in the two contexts where it has so far been studied. First, there is evidence that females discriminate kinship during violent evictions. Here individuals threaten, chase and attack same-sex conspecifics eventually expelling them from the group. Unusually, such aggression is preferentially targeted at closely related individuals, possibly because lesser relatives resist eviction to a greater extent [34]. Second, kin are often (although not always) discriminated against when mating. Inbreeding is relatively common within banded mongoose groups; both males and females can remain in their natal group for their entire lives, and the majority of breeding occurs within the group [35]. This results in 9% of pups being the product of first order inbreeding and 17% being moderately inbred [35]. Despite inbreeding occurring frequently, inbreeding avoidance occurs in the form of extra-group matings that take place during violent encounters between groups [36]. Inbreeding avoidance also occurs through non-random mating within groups, with males less likely to mate-guard closer relatives, and females breeding with lesser relatives when they evade their mate-guard and mate with a different within-group male [37]. However, it is currently unclear what mechanism may be used to identify kin in the cases of eviction or inbreeding avoidance.

In banded mongooses, social familiarity alone is unlikely to provide sufficient cues to relatedness to avoid inbreeding within social groups. For example, there is no evidence that individuals recognize their own pups or parents in as much as they do not treat them differently from other pups or adults [33]. It is therefore possible that individuals are using simple rules to avoid the closest related mates within their social group, or alternatively they may be using phenotype matching. Banded mongooses are prolific scent markers and previous studies have demonstrated that they can distinguish between individuals and the sexes via odour [38,39]. Odour therefore presents a potential mechanism by which relatedness may be detected, and inbreeding avoided.

Here, we first investigate whether banded mongooses distinguish between relatives and non-relatives based on odour. If relatedness is identifiable via odour, we predict that banded mongooses will respond differently to the odours of different individuals, depending on relatedness between the pair. Second, we investigate potential mechanisms that banded mongooses may use to distinguish between relatives and non-relatives. If relatedness is detected through assessing odour similarity, we would expect them to be able to distinguish relatedness in individuals that they are unfamiliar with. However, if they use familiarity or simple rules that require familiarity, they may only be able to distinguish the relatedness of familiar individuals.

## 2. Material and methods

### 2.1. Study population

The study was conducted on a habituated population of wild banded mongoose on Mweya peninsula in Queen Elizabeth National Park, Uganda (0°8′2^″^ S, 29°51′42^″^ E), which has been studied continuously since 1995. All mongooses are habituated to close (less than 5 m) human observation and groups are visited by trained observers approximately every 2 days meaning accurate ages, group compositions, and life history information is available. Detailed descriptions of the population, habitat and climate are provided elsewhere [30,40,41].

### 2.2. Odour collection

Banded mongooses are prolific scent markers and engage in conspicuous anal marking behaviour in addition to urinating and defecating at latrine sites [42,43]. Previous work has found that anal marking plays a key role in within-group communication and intrasexual competition [38,39,42] and chemical analyses have shown that anal gland secretions (AGS) of male and female banded mongooses differ, with females producing more chemically complex secretions [38]. For this study, we therefore focused on AGS. AGS from 49 donor males and 39 donor females from eight social groups was sampled between May and July 2014.

Animals were trapped in baited Tomahawk traps and anaesthetized using isoflurane [42] on a regular basis to refresh individual identifying marks (small shaves on the rump), to take morphometric measures and to extract AGS. Banded mongooses have two anal glands, either side of the anal opening within the anal pouch. Under anaesthesia, the anal region was cleaned with cotton wool and anal glands were expressed by applying gentle pressure. Approximately 300 µl of gland secretion was collected from each individual (150 µl from each gland) in 2 ml snap-cap glass vials (Fisher scientific) which were cleaned by soaking for several hours in methanol, air drying then soaking in detergent and warm water (1 : 1000 dilution), rinsing and allowing to air dry again. Secretions were vortexed to mix, labelled and transferred to liquid nitrogen immediately. To avoid contamination, nitrile gloves were worn and changed between individual mongooses. The examiner's fingers never came into contact with the secretion nor the top of the glass vials.

### 2.3. Odour presentations

A total of 465 presentations were conducted using 52 male and 30 female recipients from the four most habituated study groups. To assess whether banded mongooses distinguish odours on the basis of familiarity, recipients were presented, in separate trials, with odours from familiar individuals (individuals within the same social group as the recipient) and unfamiliar odours (individuals from non-neighbouring groups, thus the recipient is unlikely to have encountered these scents before). At the time of presentations, observers were blind to the relatedness between odour donor and recipient, thus removing observer and expectation bias in recording responses to odours. All donors and recipients were over 12 months of age, thus regarded as adult. No females in the study sample were pregnant or had given birth or aborted a litter within 48 h of a presentation or odour sample collection.

AGS samples were transferred to a thermos flask of ice on the morning of the presentation. Samples were fully defrosted directly before presentations, spread upon a clean ceramic tile using an autoclaved cotton swab, and presented directly to the recipient individual. Presentations were conducted when the recipient was at least 1 m away from other conspecifics and was actively foraging. Responses to the presentations were filmed using a handheld camera (Panasonic 5 Access Hybrid O.I.S, Full HD) and scored after the field session. Three measures of response to odour presentations were considered:

(1) Total marks: the number of scent marks deposited on or around the odour (within 30 cm), including urinating, depositing faeces and anal marking.
(2) Contact: the time (s) spent inspecting the odour (nose within 30 cm).
(3) Duration: the time (s) before returning to foraging behaviour, defined here as digging in topsoil/dung or vegetation or eating food items.

### 2.4. Relatedness values

While under anaesthetic, a small (approx. 2 mm) tissue sample was taken from the tip of the tail using sterile scissors, a procedure that caused little or no bleeding and did not lead to infection. DNA was extracted from tail tips by lysis with proteinase K, followed by phenol-chloroform purification or using DNA extraction kits (Qiagen™ Tissue and Blood Kit). Samples were genotyped at up to 43 microsatellite loci, following [44] or (post-2010) using multiplex PCRs (Qiagen™ Multiplex PCR Kit, UK) with fluorescent labelled forward primers following [37]. Relatedness was calculated following Lynch and Ritland [45] using the inbreedR package [46] in R v. 3.0.2.

### 2.5. Statistical analysis

Data were analysed using general linear mixed effects models (LMMs) using the lme4 package [47] within R v. 3.4.0. In each of our model sets, we included separate models for our three response variables: total marks, contact and duration. In all analyses, in order to control for repeated measurements from the same individuals and social groups, the identities and social groups of the donor and receiver were fitted as random factors. All models were run with a Gaussian error distribution and fit by restricted maximum likelihood. Model assumptions (such as normality and homogeneity of residuals and susceptibility to outliers) were checked using the ‘plot.merMod’ function in lme4. Second order interactions were included in all initial models. Non-significant terms, beginning with interactions, were sequentially removed following the backward step-wise simplification method.

1. Do banded mongooses respond differently to scents from individuals varying in relatedness?

To investigate whether behaviour in response to the scent is affected by relatedness, we fitted separate LMMs with our three response terms. We fitted relatedness between the odour donor and receiver, the sex of the donor and receiver and the age of the donor and receiver (in days) as fixed effect explanatory variables.

2. Do banded mongooses respond differently to scents from individuals that are familiar and unfamiliar?

Due to the relatedness structure within and between banded mongoose groups [48], familiar individuals (present within the same social group) are significantly more likely to be related to recipients than unfamiliar individuals (present in a different social group) (LMM, *t* = −9.161 *p*= 1.6 × 10^−18^). To investigate whether banded mongooses may be responding to the level of familiarity with an individual, rather than genetic relatedness, we constructed LMMs (one for each of our three response variables), fitting familiarity between the odour donor and receiver, the sex of the donor and receiver, and the age of the donor and receiver (in days) as fixed effect explanatory variables.

3. Do banded mongooses respond differently to scents based on relatedness, once familiarity has been controlled for?

We first split the dataset into familiar and unfamiliar presentations to control for the effect of familiarity. We then constructed separate LMMs on familiar and unfamiliar scent presentations, testing whether relatedness influenced any of our three response measures. We fitted relatedness between the odour donor and receiver, the sex of the donor and receiver, and the age of the donor and receiver (in days) as fixed effect explanatory variables.

## 3. Results

1. Do banded mongooses respond differently to scents from individuals varying in relatedness?

**Figure 1.**
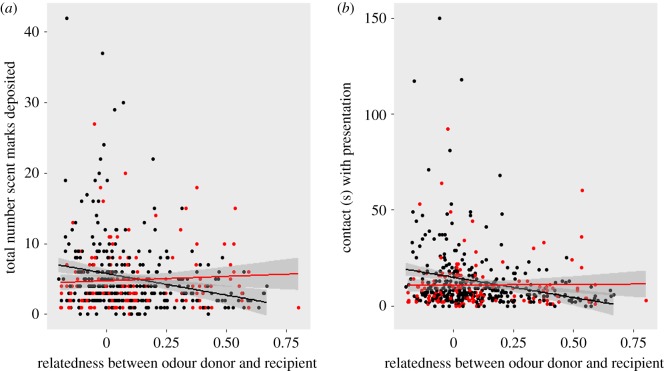
The interaction between recipient sex and their relatedness to the odour donor affects the number of scent marks deposited by the odour recipient (*a*) and the time that the recipient investigates the scent (*b*). Male recipients are represented by the black points and line, female recipients by the red points and line. Lines show regression of relatedness against marking behaviour with 95% confidence intervals.

We found that banded mongooses respond differentially to scents based on the level of relatedness of the scent donor (table 1). The sexes responded to relatedness differently, with male recipients decreasing their marking response and contact time toward odours from donors they were more related to and with females showing little (or the opposite) response to relatedness (interactions between sex and relatedness from LMMs: total marks: *t* = 2.633, *p* = 0.009, contact: *t* = 2.894, *p* = 0.004, table 1 and figure 1*a*,*b*). In addition, older recipients deposited fewer scent marks (LMM: *t* = −3.473, *p* = 0.001) and spent less time in contact with odours (LMM: *t* = −2.298, *p* = 0.022) while the odours of older individuals elicited fewer marks (LMM: *t* = −2.252, *p* = 0.025) and shorter contact times (LMM: *t* = −5.192, *p* = 3.116 × 10^−7^) and durations of interest (LMM: *t* = −4.202, *p* = 2.687 × 10^−5^).

**Table 1.** Output of LMMs testing the effect of odour donor relatedness, recipient sex, odour donor sex, recipient age and odour donor age upon response measures to presented odours. Only significant interactions are presented in the table. Non-significant fixed effects are presented alongside the *p*-values upon which they were removed from the model. Bold text highlights significant terms.

model testing	fixed effect	effect size	s.e.	*T*-value	*p*-value
total marks	intercept	9.250	0.9817	9.422
	**relatedness**	**−3.636**	**1.456**	**−2**.**497**	**0**.**009**
	**recipient sex** (**f**)	**−1.483**	**0.5397**	**−2**.**748**	**0**.**006**
	donor sex (f)			1.190	0.235
	**recipient age**	**−1.250 × 10^−3^**	**3.600 × 10^−4^**	**−3**.**473**	**0**.**001**
	**donor age**	**−5.420 × 10^−5^**	**2.406 × 10^−5^**	**−2**.**252**	**0**.**025**
	**relatedness × recipient sex** (**f**)	**6.024**	**2.288**	**2**.**633**	**0**.**009**
contact	intercept	25.68	2.098	12.241
	**relatedness**	**−16.21**	**4.586**	**−3**.**535**	**0**.**0004**
	**recipient sex** (**f**)	**−5.373**	**1.705**	**−3**.**152**	**0**.**002**
	donor sex (f)			0.836	0.404
	**recipient age**	**−2.545 × 10^−3^**	**1.108 × 10^−3^**	**−2**.**298**	**0**.**022**
	**donor age**	**−2.589 × 10^−4^**	**4.986 × 10^−5^**	**−5**.**192**	**3.116 × 10^−7^**
	**relatedness × recipient sex** (**f**)	**20.97**	**7.246**	**2**.**894**	**0**.**004**
duration	intercept	38.41	2.672	14.376
	relatedness			−1.645	0.101
	recipient sex (f)			0.903	0.367
	donor sex (f)			1.111	0.267
	recipient age			−0.007	0.994
	**donor age**	**−3.241 × 10^−4^**	**7.712 × 10^−5^**	**−4**.**202**	**2.687 × 10^−5^**

2. Do banded mongooses respond differently to scents from individuals that are familiar and unfamiliar?

**Figure 2.**
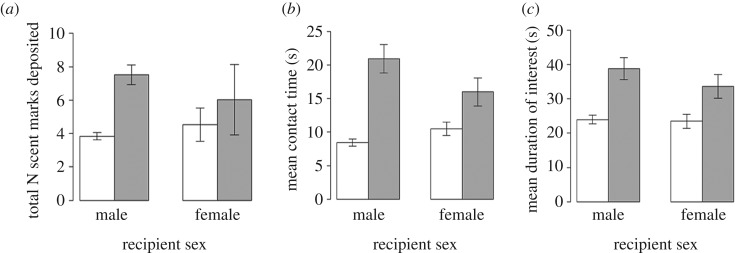
The effect of familiarity on the (*a*) number of scent marks deposited by the recipient, (*b*) the amount of time the recipient spent in contact with the scent, and (*c*) the amount of time before the recipient resumed foraging. Separate bars are shown for males and females in cases where the LMM found a significant interaction between the sex and familiarity of the odour donor. Grey bars represent unfamiliar odours and clear bars familiar odours, while error bars show standard error.

The familiarity of the scent influenced all three of our response measures (table 2 and figure 2). For contact and scent marking, there were significant interactions between the familiarity of the odour donor and the sex of the recipient. Males deposited more marks over the odours of unfamiliar individuals (LMM: *t* = −2.749, *p* = 0.006, figure 2*a*) and spent significantly longer in contact with unfamiliar than familiar odours (LMM: *t* = −2.648, *p *= 0.008, figure 2*b*). However, females did not show as strong a discriminative response. Banded mongooses of both sexes took longer to return to normal foraging behaviour after being presented with odours from unfamiliar individuals LMM: *t* = 5.507, *p* = 5.552 × 10^−8^, figure 2*c*). Older recipients deposited fewer marks (LMM: *t* = −4.485, *p* = 8.925 × 10^−6^) and spent less time in contact with odours (LMM: *t* = −3.673, *p* = 0.0002), while the age of the odour donor did not significantly influence any measure of interest in odour presentations in this analysis.

**Table 2.** Output of LMMs testing the effect of odour donor familiarity, recipient sex, odour donor sex, recipient age and odour donor age plus second order interactions upon response measures to presented odours. Only interactions with significant effects are presented within the table. Non-significant fixed effects are presented alongside *p*-values for which they were dropped from models. Bold type denotes significant effects.

model testing	fixed effect	effect size	s.e.	*T*-value	*p*-value
total marks	intercept	5.341	0.701	7.619
	**unfamiliar**	**3**.**724**	**0**.**678**	**5**.**492**	**6.146 × 10^−8^**
	recipient sex (f)	0.241	0.678	−0.460	0.646
	**donor sex** (**f**)	**0**.**942**	**0**.**411**	**2**.**293**	**0**.**022**
	**recipient age**	**−0**.**001**	**0**.**0003**	**−4**.**485**	**8.925 × 10^−6^**
	donor age			−0.108	0.525
	**familiarity × recipient sex** (**f**)	**−2**.**304**	**0**.**838**	**−2**.**749**	**0**.**006**
contact	intercept	12.037	1.720	6.998
	**unfamiliar**	**12**.**987**	**1**.**728**	**7**.**516**	**2.386 × 10^−13^**
	recipient sex (f)	0.824	1.789	0.460	0.646
	donor sex (f)	2.282	1.336	1.707	0.088
	**recipient age**	**−0**.**004**	**0**.**001**	**−3**.**673**	**0**.**0002**
	donor age			−0.745	0.457
	**familiarity × recipient sex** (**f**)	**−7**.**500**	**2**.**831**	**−2**.**648**	**0**.**008**
duration	intercept	22.432	1.998	11.229
	**unfamiliar**	**12**.**866**	**2**.**337**	**5**.**507**	**5.552 × 10^−8^**
	recipient sex (f)			−0.654	0.513
	donor sex (f)	4.459	2.294	1.944	0.052
	recipient age			−0.879	0.380
	donor age			−0.597	0.551

3. Do banded mongooses respond differently to scents based on relatedness, once familiarity has been controlled for?

**Figure 3.**
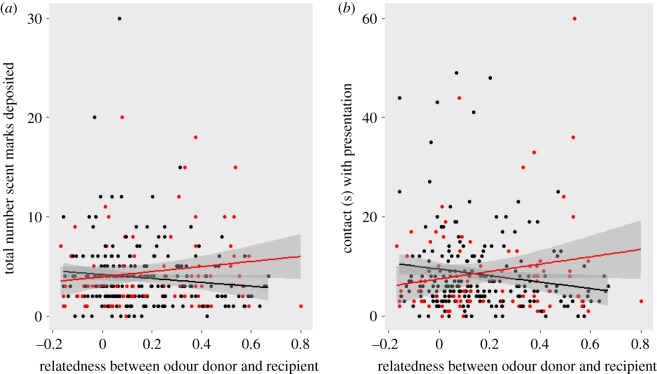
The impact of recipient sex and relatedness to the odour donor on (*a*) marking behaviour and (*b*) contact with the odour for familiar presentations. Male recipients are represented by black points and female recipients by red. Lines show regression of relatedness against marking behaviour with 95% confidence intervals.

When the odour recipient was familiar with the odour donor, we found evidence that banded mongooses responded to relatedness (table 3 and figure 3). As with the full dataset, males spent less time inspecting the sample as their relatedness to the odour donor increased, while females showed the reverse, increasing contact durations toward odours of increasing relatedness (LMM: *t* = 2.591, *p* = 0.01, figure 3). There was also a non-significant trend showing the same pattern for marking behaviour (LMM: *t* = 1.762, *p* = 0.079, figure 3).

**Table 3.** Output of LMMs testing the effect of odour donor relatedness, recipient sex, odour donor sex, recipient age and odour donor age upon response measures to familiar odours. Only significant interactions are presented in the table. Non-significant fixed effects are presented alongside *p*-values upon which they were removed from the model. Bold text highlights significant terms.

model testing	fixed effect	effect size	s.e.	*T*-value	*p*-value
total marks	intercept	29.65	3.001	9.883
	relatedness			−0.803	0.4226
	recipient sex (f)			−0.455	0.6494
	donor sex (f)			0.110	0.905
	recipient age			0.906	0.366
	**donor age**	**−6.803 × 10^−4^**	**7.912 × 10^−5^**	**−8**.**598**	**4.622 × 10^−16^**
	relatedness* × *recipient sex (f)			1.762	0.079
contact	intercept	51.322	8.090	6.344
	relatedness	−5.408	2.946	−1.836	0.067
	recipient sex (f)	−2.02	1.344	−1.502	0.134
	donor sex (f)			0.390	0.697
	recipient age			−0.530	0.596
	**donor age**	**−0**.**001**	**0**.**0002**	**−5**.**189**	**3.910 × 10^−7^**
	**relatedness × recipient sex** (**f**)	**12**.**451**	**4**.**806**	**2**.**591**	**0**.**010**
duration	intercept	29.204	6.759	4.321
	relatedness			−0.385	0.701
	recipient sex (f)			0.078	0.938
	donor sex (f)	3.918	2.258	1.735	0.297
	recipient age			−0.302	0.763
	donor age	−0.0002	0.0002	−1.045	0.084

When the odour recipient was not familiar with the odour donor, there was no effect of relatedness on any response measures (table 4). However, there was an effect of recipient sex (LMM: *t* = −2.521, *p* = 0.012), age (LMM: *t* = −3.566, *p* = 0.0004) and odour donor sex (LMM: *t* = 2.013, *p* = 0.045) on marking behaviour, and of recipient sex (LMM: *t* = −2.229, *p* = 0.027) and age (LMM: *t* = −2.341, *p* = 0.020) on the amount of time recipients spent in contact with the sample.

**Table 4.** Output of LMMs testing the effect of odour donor relatedness, recipient sex, odour donor sex, recipient age and odour age upon response measures to unfamiliar odours. Only significant interactions are presented in the table. Non-significant fixed effects are presented alongside *p*-values upon which they were removed from the model. Bold text highlights significant terms.

model testing	fixed effect	effect size	s.e.	*T*-value	*p*-value
total marks	intercept	9.522	1.082	8.798
	relatedness			−0.128	0.898
	**recipient sex** (**f**)	**−2**.**241**	**0**.**889**	**−2**.**521**	**0**.**012**
	**donor sex** (**f**)	**1**.**726**	**0**.**857**	**2**.**013**	**0**.**045**
	**recipient age**	**−0**.**002**	**0**.**0006**	**−3**.**566**	**0**.**0004**
	donor age			0.389	0.6977
contact	intercept	28.008	3.443	8.136
	relatedness			0.585	0.559
	**recipient sex** (**f**)	**−7**.**339**	**3**.**293**	**−2**.**229**	**0**.**027**
	donor sex (f)			1.053	0.294
	**recipient age**	**−0**.**005**	**0**.**002**	**−2**.**341**	**0**.**020**
	donor age			−0.066	0.947
duration	intercept	32.690	9.552	3.422
	relatedness	−9.083	27.464	−0.331	0.741
	recipient sex (f)	−9.696	6.019	−1.611	0.109
	donor sex (f)	7.176	6.392	1.122	0.264
	recipient age	−0.0009	0.004	−0.223	0.824
	donor age	0.0002	0.0002	1.000	0.319

## 4. Discussion

We found that banded mongooses are able to distinguish between odours based on relatedness. Such discrimination appears in part due to differential responses to the scents of familiar (within-group) and unfamiliar (non-neighbouring group) individuals, with heightened responses being shown towards odours that are unlikely to have been encountered previously, particularly for male recipients. As an individual's social group contains close kin, while a social group that has not been encountered previously is very unlikely to contain close relatives [48], group-membership is usually a reliable indicator of relatedness in the banded mongoose and in other group-living mammals [8,10].

When presenting unfamiliar odours to banded mongooses, we found no evidence that individuals differ in their behavioural responses to odours based on relatedness. These results are in contrast to those from a scent-presentation study on the closely related meerkat [26], where dominant females investigated unfamiliar scents for longer when they were from related subordinate males. It is currently unclear why these two species should differ in their behavioural responses to unfamiliar relatives, but it is possible that meerkats are more likely to encounter unfamiliar potential mates as subordinate males often rove across the territories of other social groups in search of mating opportunities [49]. This roving behaviour is rare in banded mongooses, and the majority of breeding occurs either within the social group (82% paternities) or between neighbouring social groups (16% paternities), with matings between non-neighbouring groups being very rare (2% paternities) [36]. The lack of opportunities for mating with unfamiliar individuals may therefore reduce the benefits of distinguishing between unfamiliar individuals on the basis of relatedness in banded mongooses. Furthermore, in our study, just 9% of unfamiliar individuals had *r* > 0.125 (cousin level) and 1.8% had *r* > 0.25 (half-sibling level), while among familiar individuals, 48% were related by at least 0.125 and 32% by at least 0.25. The lack of behavioural discrimination of relatedness in unfamiliar trails may thus be simply explained by the fact that unfamiliar individuals are rarely related to the recipient.

Interestingly, we found that animals discriminated relatedness when presented with familiar odours (from fellow group-members). Here, males spent longer investigating scents from unrelated individuals, while females spent longer investigating scents from closer relatives. This raises the possibility that banded mongooses are using a learned cue to identify relatives from within their social group. Our experiment is not able to determine whether such cues are obtained directly from the odour (such as via phenotype matching), or whether they are gained from elsewhere (for example from behaviour) but are associated with the odour of the corresponding individual through learning.

In many group-living species, simple rules that rely on familiarity are used to identify likely kin [10]. For example, individuals may treat all individuals who were familiar to them shortly after birth/hatching as close kin, regardless of genetic relatedness, a strategy that appears common in cooperatively breeding birds [9]. However, such a strategy is unlikely to be effective in the banded mongoose as the alloparental care provided by group-members is not biased towards kin [33]. In other cooperative breeders, non-relatives can often be identified by their immigration status; immigrants are usually unrelated to other group-members, and natal individuals often refrain from breeding unless immigrants are present within the group (e.g. Damaraland mole-rats *Cryptomys damarensis* [17] and acorn woodpeckers *Melanerpes formicivorus* [50]). However, in our study population of banded mongooses, immigration into established groups is practically absent and individuals of both sexes remain philopatric beyond sexual maturity, with over 80% of individuals spending their entire lives living and breeding in their natal group [41]. Therefore, immigration is likely to be an ineffective way of assessing relatedness, at least for natal individuals. In contrast, group-founders may be able to use immigration status to identify unrelated mates. Banded mongoose groups form when a cohort of single-sex dispersers from one group either joins with a cohort of opposite-sex dispersers from a different social group or ousts all same-sex group-members from an existing group [51]. Therefore, in a newly formed group, males and females are unrelated to each other [48]. Group founders are therefore likely to retain the ability to identify other (unrelated) founders. Indeed, female group founders are less likely to breed with extra-group males than natal females [35], presumably as female founders have reliable access to unrelated male founders from within the group. However, it is not possible that the patterns we found in our data were driven by differences in responses by group founders as none of the social groups where scent presentations were conducted contained surviving group founders.

Finally, it is possible that genetic relatedness assessment through odour similarity does in fact occur in banded mongooses but is restricted to facilitating within-group kin recognition. The majority of matings happen within groups, and relatedness to other group-members varies widely; median *r* = 0.12, inter-quartile range = 0.01 to 0.32, compared to median *r* = −0.03, inter-quartile range = −0.09 to 0.03 between unfamiliar individuals. It is therefore likely to benefit individuals to discriminate between group members based on relatedness [37,48]. Indeed, discrimination based on olfactory cues may explain patterns of inbreeding avoidance in this species, whereby males are less likely to mate-guard a female as relatedness between the pair increases, and females that evade their guard breed with males of lower relatedness [37]. Such a mechanism may also explain patterns of aggression during violent mass evictions, where dominant females preferentially attack more closely related female subordinates [34]. It is possible that habituation-dishabituation trails, rather than current methodologies, would be better able to reveal the banded mongooses' ability to discriminate the odours of unfamiliar individuals based on relatedness [24]. For example, we expect that odours from individuals that are similarly related to the focal individual would be perceived to be more similar to each other than odours from individuals with large differences in relatedness to the focal animal. Furthermore, future studies analysing the chemical composition of scents, for example through gas chromatography–mass spectrometry (GCMS) of anal gland secretions, may also be able to shed light on the possibility of genetic relatedness assessment, as chemical cues to relatedness have been identified in other species where mates appear to be selected on the basis of genetic relatedness [52,53].

If chemical cues to relatedness exist in banded mongoose odours, it seems unlikely that they allow sufficient resolution of kinship to distinguish between close relatives such as one's own offspring and the offspring of siblings or cousins. Birth is highly synchronized in this species, and offspring are combined into a communal litter shortly after birth, from which point they are raised communally [31]. This appears to result in a lack of behavioural cues to kinship within groups as each adult is likely to be equally familiar with all pups. When cues do exist, for example when females give birth out of synchrony (or when females are experimentally given contraceptives), pregnant females can identify newborn pups as not being their own [31,32]. Dominant females therefore kill the pups of subordinates, which reduces competition for resources with their own pups [31,32,54]. If it were possible to definitively identify one's own pups using odour, it seems likely that infanticide by dominant females would be more common, even when births are synchronous. It is possible that the fitness costs of mistakenly killing one's own pups is too great to risk based on an imperfect kin discrimination system. Furthermore, it may benefit pups to remain anonymous when under the threat of infanticide by non-relatives. It is therefore possible that pups have evolved ways in which to ‘mask’ their genetic identity with regards to olfactory communication [33].

An additional finding of our study was that males and females respond differently to odours varying in relatedness and familiarity. Males displayed a stronger response to unfamiliar scents than females, spending more time investigating the scent and depositing more scent marks on and around unfamiliar scents. The greater role that males play in territory defence in this species may explain this difference [55]. When presented with scents from familiar individuals, males spent longer investigating scents as relatedness decreased, while females showed the opposite pattern. However, it is currently unclear why males and females might respond to within-group relatedness differently. We also found that response to odours differed depending on the age of the donor, with the odour of older individuals receiving weaker responses, at least among familiar individuals. This may occur because more dominant individuals (usually the older individuals in the group [44]) scent mark at higher rates [39], hence group-members may become habituated to these odours and therefore show a reduced response. This may also explain why a response to age was not seen between unfamiliar individuals where there was no opportunity to habituate to the odours prior to the experiment. Finally, we found that responses to unfamiliar odours varied based on sex, with females receiving greater marking responses. Here, it is likely that individuals are responding directly to chemical differences between the sexes as a previous study has demonstrated sex-differences in odour profiles [38].

## 5. Conclusion

We investigated the use of olfactory cues in the discrimination of kin in a cooperative carnivore where offspring are reared communally. We found that banded mongooses are able to discriminate between familiar and unfamiliar individuals based on odour alone, and that individuals respond to odours of their own group-members differently based on genetic relatedness between themselves and the odour donor. However, we found no evidence for discriminatory responses to odours based upon their relatedness among unfamiliar individuals. It is likely that, for this species, assessment of relatedness is more beneficial at the within-group rather than between-group level due to the high degree of variation in relatedness within social groups, where most mating opportunities arise. Our experiment was not able to determine whether banded mongooses were simply unable to discriminate odours of unfamiliar individuals based on relatedness, or whether they were able to discriminate but did not alter their behavioural responses to the odour. Furthermore, as most unfamiliar individuals were unrelated to odour recipients, there may not have been sufficient variability in relatedness between unfamiliar individuals to detect a response. Future studies investigating the chemical composition of banded mongoose scents will be able to further investigate the possibility that information regarding relatedness can be conveyed through odours themselves, while detailed investigations of the relatedness structure of banded mongoose groups may reveal factors that vary with relatedness that could be used as non-olfactory indicators of kinship.

## Supplementary Material

Banded mongoose scent presentation data

## Data accessibility

Data are available at Dryad: https://doi.org/10.5061/dryad.vt1qd42 [56].
